# Identification of Ferroptosis-Related Prognostic Signature and Subtypes Related to the Immune Microenvironment for Breast Cancer Patients Receiving Neoadjuvant Chemotherapy

**DOI:** 10.3389/fimmu.2022.895110

**Published:** 2022-05-04

**Authors:** Yuhao Xu, Yaoqiang Du, Qinghui Zheng, Tao Zhou, Buyun Ye, Yihao Wu, Qiuran Xu, Xuli Meng

**Affiliations:** ^1^ The Second Clinical Medical College, Zhejiang Chinese Medical University, Hangzhou, China; ^2^ General Surgery, Cancer Center, Department of Breast Surgery, Zhejiang Provincial People’s Hospital (Affiliated People’s Hospital, Hangzhou Medical College), Hangzhou, China; ^3^ Laboratory Medicine Center, Department of Transfusion Medicine, Zhejiang Provincial People’s Hospital (Affiliated People’s Hospital, Hangzhou Medical College), Hangzhou, China; ^4^ Hangzhou Medical College, Hangzhou, China; ^5^ College of Pharmacy, Zhejiang University of Technology, Hangzhou, China; ^6^ Laboratory of Tumor Molecular Diagnosis and Individualized Medicine of Zhejiang Province, Zhejiang Provincial People’s Hospital (Affiliated People’s Hospital, Hangzhou Medical College), Hangzhou, China

**Keywords:** breast cancer, ferroptosis, relapse-free survival, neoadjuvant chemotherapy, immune microenvironment

## Abstract

**Purpose:**

To identify molecular clusters associated with ferroptosis and to develop a ferroptosis-related signature for providing novel potential targets for the recurrence-free survival and treatment of breast cancer.

**Methods:**

Ferroptosis-related gene (FRG) signature was constructed by univariate and multivariate Cox regression and least absolute shrinkage and selection operator (LASSO). Receiver operating characteristic curves, Kaplan–Meier survival analysis, principal component analysis, and univariate and multivariate Cox regression analyses in the training and test cohorts were used to evaluate the application of this signature. Quantitative reverse transcriptase–PCR (qRT-PCR) was employed to detect the expression of FRGs in the model. Furthermore, the correlations between the signature and immune microenvironment, somatic mutation, and chemotherapeutic drugs sensitivity were explored.

**Results:**

Internal and external validations affirmed that relapse-free survival differed significantly between the high-risk and low-risk groups. Univariate and multivariate Cox regression analyses indicated that the riskScore was an independent prognostic factor for BRCA. The areas under the curve (AUCs) for predicting 1-, 2-, and 3-year survival in the training and test cohorts were satisfactory. Significant differences were also found in the immune microenvironment and IC50 of chemotherapeutic drugs between different risk groups. Furthermore, we divided patients into three clusters based on 18 FRGs to ameliorate the situation of immunotherapy failure in BRCA.

**Conclusions:**

The FRG signature functions as a robust prognostic predictor of the immune microenvironment and therapeutic response, with great potential to guide individualized treatment strategies in the future.

## Introduction

Breast cancer has surpassed lung cancer as being the most commonly diagnosed cancer with approximately 2.3 million new cases in 2020, accounting for 11.7% of all new cancer cases (1). Another scary truth is the drop in average onset age (2). Because breast cancer is a highly heterogeneous systemic disease, advancements in therapy are particularly crucial (3).

Neoadjuvant chemotherapy (NAC) is seen as the standard and first-line treatment for locally advanced breast cancer (4, 5), which not only is beneficial to breast-conserving surgery but also can detect tumor sensitivity to anticancer therapy for locally advanced breast cancer (6), and it could also be employed as a bridge to other therapies (7, 8). Anthracyclines and taxanes serve as the backbone of NAC regimens and are widely used clinically (9).

Ferroptosis is an emerging form of programmed cell death featured by the iron-dependent accumulation of lipid reactive oxygen species (ROS) of metabolic dysfunctions, iron accumulation, and antioxidant vulnerability (10–12). Accumulating evidence showed that the role of ferroptosis in carcinogenesis, progression, and chemoresistance had made progress. Fascin regulates SLC7A11 stability to induce ferroptosis (13). Renovation of SLC7A11 rescues miR-5096-mediated ferroptosis and antitumor effects of breast cancer (14). Ferroptosis-related gene (FRG) GPX4 promotes chemoresistance in nasopharyngeal carcinoma (15). Bufotalin induces ferroptosis by facilitating the ubiquitination and degradation of GPX4 in non-small cell lung cancer cells (16). *Via* ferroptosis, ETS1/miR-23a-3p/ACSL4 axis stimulates sorafenib resistance in HCC (17).

Ferroptosis has the characteristics of inhibiting chemoresistance and enhancing antitumor immunity (18), which may be a potential strategy to overcome the drug resistance mechanism of traditional cancer treatments (12). Previous studies prove the feasibility of ferroptosis-related prognostic markers to predict overall survival and immune characteristics. FRG signatures were constructed to predict overall survival in lung adenocarcinoma (19), colorectal cancer (20), and pancreatic adenocarcinoma (21). However, as far as we are aware, studies focusing on the correlation of ferroptosis with biochemical recurrence and antitumor immunology of BRCA were rather limited. Thus, it is an urgent need to discover a robust biomarker to predict relapse-free survival (RFS) in BRCA.

In this study, we constructed an FRG prognostic signature and identified three ferrClusters in predicting the RFS internally and externally, exploring the status of immune infiltrates and drug sensitivity of BRCA patients receiving NAC for guiding clinical practice. This signature may also serve as a novel and robust prediction tool for evaluating whether BRCA patients can benefit from immunotherapy.

## Methods

### Data Acquisition and Processing

Open expression matrix of mRNA (FPKM values) and clinical files of BRCA samples were downloaded from The Cancer Genome Atlas (TCGA) database. Datasets GSE25055 in the Gene Expression Omnibus (GEO) database were used to acquire RNA-sequencing (RNA-Seq) and clinical data of BRCA patients receiving NAC as a training cohort and GSE16446 and GSE25065 as test cohorts. Gene expression file of GSE25055 and GSE25065 was collected using platform GPL96 [HG-U133A] Affymetrix Human Genome U133A Array, and GSE16446 using platform GPL570 [HG-U133_Plus_2] Affymetrix Human Genome U133 Plus 2.0 Array. Batch effects and other unwanted variations in high-throughput experiments were eliminated using the “combat” function in the “sva” package (22) in R 4.1.1. Copy number variation (CNV) data were collected from the University of California, Santa Cruz (UCSC) website.

### Construction of the Ferroptosis-Related Signature for Predicting Recurrence-Free Survival

FRGs including 150 drivers, 109 suppressors, and 123 markers were collected from FerrDb (19, 23, 24). A univariate Cox proportional hazards regression analysis was conducted to filtrate prognostic FRGs in the GSE25055 cohort with p < 0.05 considered to be statistically significant using the “coxph” function. Subsequently, with the help of the “cv.glmnet” function, the least absolute shrinkage and selection operator (LASSO) was performed for the dimension reduction and K-fold cross-validation, which was multiplied by ten, and the optimal parameter was the λ value that corresponded to the lowest deviation. The optimal penalty parameter was defined as the value within one SD of the minimum cross-validated partial likelihood deviance to obtain the best model. The proteins with non-zero regression coefficients were chosen for subsequent multivariate Cox regression analyses. The LASSO regression model was as follows:


$$\text{riskScore}=\sum_{N=A,B\ldots n}\text{Coefficient of gene N}\times \text{Expression value of gene N}$$

### Validation of the Prognostic Signature

First, in the GSE25055 dataset, the Kaplan–Meier (K-M) survival analysis using the “Surv” function in the “survival” package and univariate and multivariate Cox regression analyses between gene expression and clinical characters using the “coxph” function in the “survival” package were performed to certify that riskScore served as an independent predictor in predicting recurrence-free survival (RFS). Principal component analysis (PCA) using the “prcomp” function was used to visualize sample distribution. Receiver operating characteristic (ROC) using the “timeROC” package was done, and area under the curve (AUC) plots were generated for the 1-year, 2-year, and 3-year survival rates to assess the sensitivity and specificity of the prognostic model. Then, the prognostic signature was validated in the GSE25065 and GSE16446 datasets *via* the above methods.

### Cell Culture

Normal breast epithelial cell line MCF-10A and the epithelial BRCA cell lines MCF-7, T47D, MDA-MB-231, MDA-MB-468, and BT-549 were acquired from the American Type and Culture Collection (ATCC; Manassas, VA, USA). MDA-MB-231 and BT-549 cells were cultured in Dulbecco’s Modified Eagle’s Medium (DMEM) (ATCC; Manassas, VA, USA) supplemented with 10% fetal bovine serum (HyClone, Logan, UT, USA) and 1% antibiotic (100 IU/ml of penicillin and 100 µg/ml of streptomycin; HyClone, Logan, UT, USA). MCF-10A cells were cultured in DMEM/F12 medium supplemented with 20 ng/μl of epidermal growth factor, insulin, hydrocortisone, non-essential amino acid (NEAA), 5% horse serum (HS), and 1% penicillin/streptomycin (P/S) solution (Procell, Wuhan, China). MCF-7 and MDA-MB-468 were cultured in Minimum Essential Medium (MEM) (Gibco BRL, Grand Island, NY, USA) supplemented with 10% fetal bovine serum (HyClone, Logan, UT, USA) and 1% antibiotic (100 IU/ml of penicillin and 100 µg/ml of streptomycin; HyClone, Logan, UT, USA). T-47D cells were cultured in Roswell Park Memorial Institute (RPMI) 1640 (HyClone, Logan, UT, USA) with 10% fetal bovine serum (HyClone, Logan, UT, USA). All the cell lines were incubated at 37°C, with a humidified atmosphere of 5% CO_2_.

### Quantitative Reverse Transcriptase–PCR

Total RNAs were isolated from cells using the TRIzol reagent (Invitrogen, Carlsbad, CA, USA). PrimeScript™ RT reagent Kit (Takara, Maebashi, Japan) was employed to reverse transcribe into cDNA following the manufacturer’s protocol. Then SYBR Green PCRMaster Mix (Applied TaKaRa, Otsu, Japan) was used to conduct Real-time PCR on Applied Biosystems 7500 Fast Real-Time RCR System (Applied Biosystems, Foster City, CA, USA). The primers of FRGs for qRT-PCR utilized in this research were as follows:

**Table:** 

Primer name	Primer sequence (5′ to 3′)
SLC7A5-F	GTGGACTTCGGGAACTATCACC
SLC7A5-R	GAACAGGGACCCATTGACGG
ACO1-F	CGCAGCACAAGAACATAGAAGT
ACO1-R	CATTGCAGCAAAGTCAACCAC
ENPP2-F	TCGCTGTGACAACTTGTGTAAG
ENPP2-R	CCAATGCGACTCTCCTTTGC

### Drug Sensitive Analysis

With the use of the “pRRophetic” package, the half-maximal inhibitory concentration (IC50) of BRCA patients was calculated on Genomics of Drug Sensitivity in Cancer (GDSC) (25) (https://www.cancerrxgene.org/) based on the given gene expression profiles in these datasets to evaluate the drug sensitivities (26–28).

### Immune Infiltration Analyses

The CIBERSORT algorithm was used to explore the proportion of different types of immune cells in BRCA patients using CIBERSORT R script v1.04 (29–31). Based on the expression level of immune cell-related genes, the ESTIMATE algorithm was conducted to calculate the stromal score (SS), estimate score (ES), and immune score (IS) (the SS represents the level of stroma content in a tumor; the IS reflects the infiltration of immune cells in a tumor; the estimated score infers tumor purity) among the high- and low-risk groups using the “estimate” package (32). Single-sample gene set enrichment analysis (ssGSEA) was performed to calculate scores for antitumor immunity and protumor suppression for each sample (33) using “GSEABase” and “GSVA” packages.

### Consensus Clustering Analyses for Identifying BRCA Subtypes

Consensus clustering based on Euclidean distance and Ward’s linkage was performed for hierarchical clustering to identify different subtypes using the “ConsensusClusterPlus” package and repeated the procedures 1,000 times to guarantee the stability of the classification (34). In consideration of a high consistency of clusters, a low coefficient of variation, and no significant increase in the CDF curve, the optimum cluster number could be determined (35).

### Statistical Analysis

Correlation coefficients were calculated by Spearman’s and distance correlation analyses. For comparison of more than two groups, the Kruskal–Wallis and one-way ANOVAs were chosen as non-parametric and parametric methods, while Wilcoxon’s t-test was used for two groups. Student’s t-test was used to explore the statistical significance of quantitative data. The K-M and log-rank tests were employed to confirm the significance of prognostic differences (22). R 4.1.1 software was the main tool to conduct the statistical analysis. For all statistical results, a p-value of <0.05 was considered to be statistically significant.

## Results

### Construction of the Ferroptosis-Related Signature Associated With Recurrence-Free Survival

**Figure 1:**
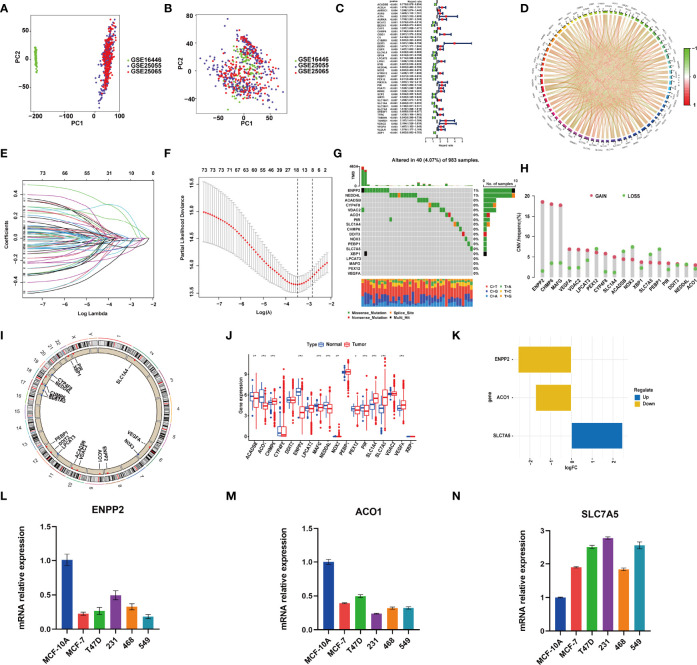
RNA-sequencing (RNA-Seq) data of training and test cohorts before **(A)** and after **(B)** removing batch effects. **(C)** The hazard ratio (HR) and p-value of selected ferroptosis-related genes (FRGs) using the univariable Cox HR regression (criteria: p-value <0.01). **(D)** Expression interaction of the 76 FRGs in BRCA. The lines connecting the FRGs show how they are correlated with each other, with positive associations in red and negative associations in green. **(E)** The least absolute shrinkage and selection operator (LASSO) Cox analysis identified 18 FRGs most related to prognostics. **(F)** The 10-round cross-validation determined the optimal values of the penalty parameter. **(G)** In all, 40 of 983 (4.07%) BRCA patients experienced 18 FRG genetic alterations. **(H)** Copy number variation (CNV) mutation frequency of the 18 FRGs. This column represents the frequency of change. Deletion frequency is represented by green dots, while amplification frequency is represented by pink dots. **(I)** The location of the 18 FRGs in chromosomes. Blue point represents the genes that mainly had CNV deletion; red point represents the genes that mainly had CNV amplification. **(J)** Expression of the 18 FRGs in normal tissues and BRCA tissues. Genes with red color represent the differentially expressed genes. **(K)** The value of logFC of the 18 FRG genes. **(L–N)** qRT-PCR results showed the expression value of the three FRGs in the normal breast and five breast cancer cell lines. *, P < 0.05; **, P < 0.01; ***, P < 0.001.

GSE25055 dataset was used as a training cohort; meanwhile, GSE25065 and GSE16446 datasets were used as test cohorts. Batch effects were removed for further study (**Figures 1A, B**). First, we performed a univariate Cox regression analysis in GSE25055. Among 382 FRGs retrieved from the FerrDB database, 76 FRGs were identified to be associated with RFS, with the standard of p < 0.01 (**Figure 1C**). Pearson’s correlation analysis revealed a correlation among these genes (**Figure 1D**). Then, LASSO regression analysis was used to establish the FRGs prognostic signature (**Figures 1E, F**):


$$\begin{array}{l} \text{riskScore }=\text{ ACADSB expression}\times \left(-0.020510043\right)\\ \;+\text{ACO}1\text{ expression}\times \left(0.007304261\right)\\ \;+\text{ CHMP}6\text{ expression}\times \left(-0.086718343\right)\\ \;+\text{CYP}4\text{F}8\text{ expression}\times \left(-0.02261864\right)\\ \;+\text{DDIT}3\text{ expression}\times \left(0.289634547\right)\\ \;+\text{ENPP}2\text{ expression}\times \left(0.002930772\right)\\ \;+\text{LPCAT}3\text{ expression}\times \left(-0.054795656\right)\\ \;+\text{MAFG expression}\times \left(0.015720631\right)\\ \;+\text{NEDD}4\text{L expression}\times \left(-0.154434979\right)+\text{NOX}3\\ \;\times \left(-0.152413703\right)+\text{PEBP}1\times \left(-0.116654913\right)\\ \;+\text{PEX}12\text{ expression}\times \left(0.021389428\right)+\text{PIR}\\ \;\times \left(0.005001444\right)+\text{SLC}1\text{A}4\times \left(-0.094069987\right)\\ \;+\text{SLC}7\text{A}5\times \left(0.165365881\right)+\text{VDAC}2\\ \;\times \left(0.247634891\right)+\text{VEGFA}\times \left(0.029115114\right)\\ \;+\text{XBP}1\times \left(-0.077572219\right) \end{array}$$

### Landscape of Gene Mutations and Expression in Ferroptosis-Related Genes in the Model in BRCA

Genomic mutations were common in these genes with 40 (4.07%) of 983 patients having experienced genetic changes, and a mutation frequency of 1% was observed in ENPP2 and NEDD4L (**Figure 1G**). We also found that CNV is prevalent among the 18 FRGs. ENPP2, CHMP6, MAFG, VEGFA, VDAC2, LPCAT3, CYP4F8, SLC1A4, XBP1, PIR, and ACO1 showed copy number amplification, while deletion happened in the other FRGs (**Figure 1H**). The location of the 18 FRGs in human chromosomes could be seen in **Figure 1I**. The result of differential analysis in normal breast tissue and tumor tissue showed that ACO1, CHMP6, ENPP2, MAFG, NEDD4L, PIR, SLC1A4, SLC7A5, and VEGFA had significant differential expression in breast cancer with p-value <0.001; ACADSB and NOX3 with p-value <0.01; and PEX12 with p-value <0.05 (**Figure 1J**). SLC7A5 was seen as a significantly upregulated gene, while ENPP2 and ACO1 were seen as significantly downregulated genes with |log FC| > 1 (**Figure 1K**). The result of RT-PCR provided strong support for our conclusion (**Figures 1L–N**). As described above, FRGs had significant heterogeneity of genetic variation and transcriptomic alteration landscape in BRCA patients, which played an important part in regulating the happening, aggravation, and prognosis of BRCA.

### External Validation of the Ferroptosis-Related Gene Model

**Figure 2:**
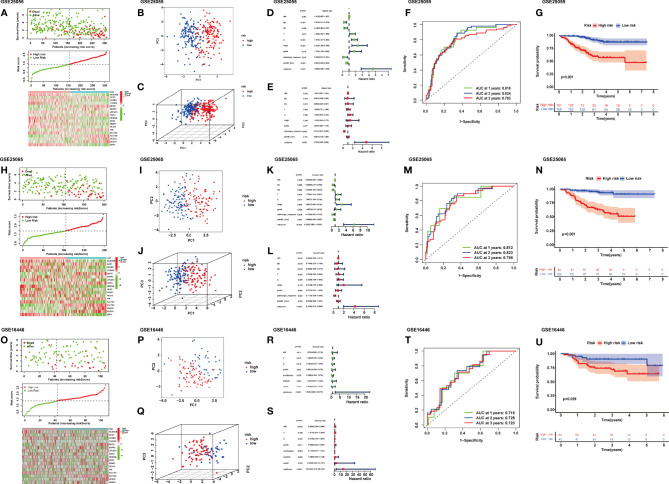
Distribution of riskScore, scatterplot, and heatmap in the high-risk group and the low-risk group in GSE25055 **(A)**, GSE25065 **(H)**, and GSE16446 **(O)**. PCA (principal component analysis) for BRCA based on the riskScore in GSE25055 **(B, C)**, GSE25065 **(I, J)**, and GSE16446 **(P, Q)**. **(D)** Univariate and **(E)** multivariate Cox regression analyses of age, estrogen receptor (ER), progesterone receptor (PR), T, N, stage, grade, pathologic response, pam50 classification, and riskScore in GSE25055. **(K)** Univariate and **(L)** multivariate Cox regression analyses of age, ER, PR, T, N, stage, grade, pathologic response, pam50 classification, and riskScore in GSE25065. **(R)** Univariate and **(S)** multivariate Cox regression analyses of age, T, N, grade, her2-fish, top2atri, erbb2, and riskScore in GSE16446. Time-dependent receiver operating characteristic (ROC) curves for predicting 1-, 2-, and 3-year RFS in GSE25055 **(F)**, GSE25065 **(M)**, and GSE16446 **(T)**. Kaplan–Meier curves of the high- and low-risk subgroup patients in GSE25055 **(G)**, GSE25065 **(N)**, and GSE16446 **(U)**.

After the riskScore of each patient based on the risk model was calculated, we divided patients into the high- and low-risk groups with the standard median score in GSE25055 (**Figure 2A**). With the use of the median of GSE25055, patients in GSE25065 (**Figure 2H**) and GSE16446 (**Figures 2H, O**) were separated into the high-risk and low-risk groups in the same manner. The result of PCA showed significant heterogeneity between high-risk and low-risk patients in GSE25055 (**Figures 2B, C**), GSE25065 (**Figures 2I, J**), and GSE16446 (**Figures 2P, Q**), which certified the superior discrimination of the FRG model. For the purpose of exploring whether the signature could represent its prognostic value independently of other clinical factors, we conducted univariate and multivariate Cox regression analyses in the training and test cohorts. In univariate analyses, this risk score was able to independently predict survival outcomes in GEO cohorts (GSE25055, hazard ratio (HR) = 4.690, p < 0.001; GSE25065, HR = 6.350, p < 0.001; GSE16446, HR = 7.648, p < 0.001) (**Figures 2D, K, R**). The same conclusion could be drawn in multivariate analyses (**Figures 2E, L, S**). The results revealed that riskScore and pathologic response served as independent factors affecting receiving NAC BRCA patients’ prognosis. The AUCs of the time-dependent ROC curves at 1, 2, and 3 years were 0.818, 0.824, and 0.783 in GSE25055 (**Figure 2F**); 0.812, 0.824, and 0.783 in GSE25065 (**Figure 2M**); and 0.715, 0.725, and 0.723 in GSE16446 (**Figure 2T**). The AUCs in different years and cohorts were relatively high compared with those of other published literature, which suggested high sensitivity and specificity of the signature for predicting RFS. The K-M survival curve showed that patients in the high-risk group had a higher recurrence rate than those in the low-risk group using log-rank tests with p < 0.001 (**Figure 2G**), p < 0.001(**Figure 2N**), p = 0.029 (**Figure 2U**). Ferroptosis is a recently recognized form of regulated cell death that is characterized by lipid peroxidation, which mediates cell death in breast cancer. Among genes in our signature, ferroptosis driver genes such as NOX3 and PEBP1 had negative coefficients, while ferroptosis suppressor genes such as PIR and VDAC2 had positive coefficients. Therefore, high riskScore indicated that ferroptosis was suppressed in breast cancer, which might imply a worse prognosis.

### Clinicopathological Parameter Relevance Analysis

**Figure 3:**
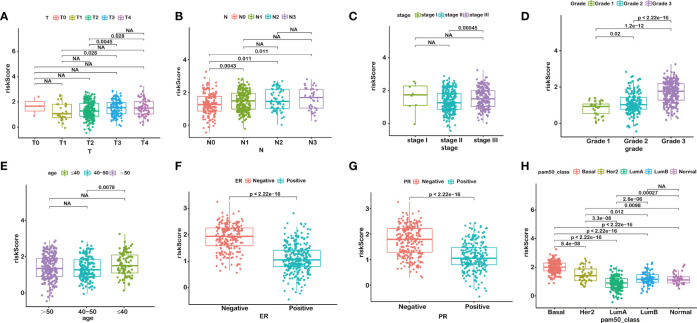
RiskScore is correlated with clinicopathological features of BRCA. T stage **(A)**, N stage **(B)**, AJCC-stage **(C)**, Grade **(D)**, age **(E)**, ER status **(F)**, PR status **(G)**, PAM50 subtypes **(H)**. NA, P>0.05.

We further anatomized the association between riskScore and clinical parameters of BRCA patients. The detailed results depicted that the riskScore had a positive correlation with T stage, N stage, American Joint Committee on Cancer (AJCC) stage, and grade (**Figure 3**). BRCA patients with higher T, N, AJCC stage, and grade, combined with lower age, and negative status of progesterone receptor and estrogen receptor seemed to have higher riskScore, indicating a higher incidence rate of relapse, which was consistent with the conclusions of current accumulated literature. In other words, the results implied that the riskScore had a correlation with clinicopathological parameters.

### Chemotherapeutic Response Analysis

**Figure 4:**
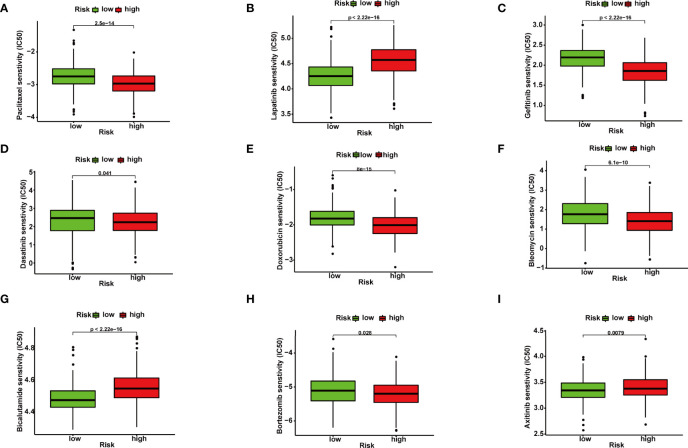
Correlation between ferroptosis-related gene (FRG) signature and drug sensitivity. Box plots for estimated IC50 of drugs between high- and low-risk BRCA patients. Paclitaxel **(A)**, Lapatinib **(B)**, Gefitinib **(C)**, Dasatinib **(D)**, Doxorubicin **(E)**, Bleomycin **(F)**, Bicalutamide **(G)**, Bortezomib **(H)**, Axitinib **(I)**.

In order to improve the therapeutic benefit of BRCA patients from neoadjuvant therapy, we further explored whether FRG signature could predict the sensitivity to several chemotherapy drugs widely used in BRCA between two groups. According to the results calculated based on the GDSC database, IC50 values of chemotherapy drugs covering axitinib, bicalutamide, bleomycin, bortezomib, dasatinib, doxorubicin, gefitinib, lapatinib, and paclitaxel were evaluated. Compared with the low-risk group, IC50 values of paclitaxel, gefitinib, doxorubicin, bleomycin, and bortezomib were lower in the high-risk groups, which indicated that high-risk patients were more sensitive to these drugs (**Figures 4A–I**). The above results demonstrated that the riskScore had potential predictive value for chemotherapy and targeted therapy in breast cancer.

### Comprehensive Analysis Between Ferroptosis-Related Gene Signature and Immune Microenvironment

**Figure 5:**
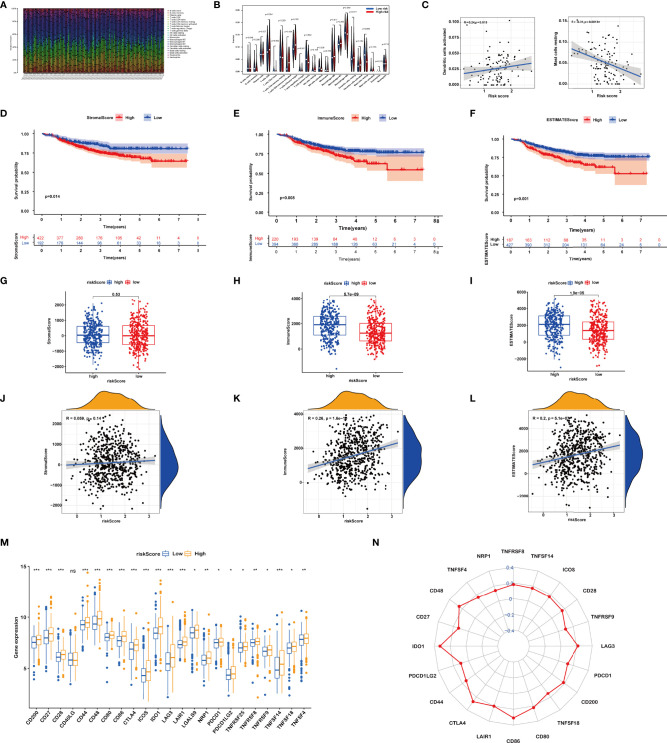
**(A)** Relative percent of different immune cells in each sample. **(B)** Different immune cell contents in low-risk and high-risk patients. **(C)** Correlation between immune cells and riskScore. **(D–F)** Kaplan–Meier curves of the high and low stromal score (SS), immune score (IS), and estimate score (ES) group patients. **(G–L)** Correlation between riskScore and SS, IS, and ES. **(M, N)** Comparisons of the expression levels of immune checkpoints between two groups. ns, P>0.05. *, P < 0.05; **, P < 0.01; ***, P < 0.001.

We calculated the constitution of tumor-infiltrating immune cells in BRCA through the CIBERSORT algorithm (**Figure 5A**). Compared with the low-risk groups, the proportion of resting mast cells was lower in the high-risk groups (**Figures 5B, C**).

Then, the IS, SS, and ES of patients were evaluated using the ESTIMATE algorithm. Based on the optimum cutoff value of ISs or SSs respectively, BRCA patients were divided into the high and low IS/SS/ES groups. The K-M curves showed that patients with high IS/SS/ES exhibited significantly worse RFS as compared to the ones with low IS/SS/ES (**Figures 5D–F**). We further explored the relationships between the IS/SS/ES and riskScore. The result of Wilcoxon’s rank-sum test displayed that there is no significant difference between the high-risk and low-risk groups in SS (p = 0.53, **Figure 5G**) but significant in IS (p = 5.7e−09, **Figure 5H**) and ES (p = 1.9e−05, **Figure 5I**). Pearson’s correlation analysis showed that riskScore was positively associated with IS (R = 0.26, p = 1.6e−10, **Figure 5K**) and ES (R = 0.2, p = 5.1e−07, **Figure 5L**). However, the riskScore was not significantly correlated to the SS (P = 0.14, **Figure 5J**)

Furthermore, we dissected the role of riskScore in immune checkpoint blockade (ICB) treatment. We noticed that the expression levels of all immune checkpoints were significantly higher in the high riskScore group (**Figures 5M, N**). Taken together, the prognostic signature could predict the potential response to immunotherapy in BRCA patients, which provided guidance on whether or what to use for immunotherapy in clinical practice.

### Identification of Three Consensus Clustering Subtypes

**Figure 6:**
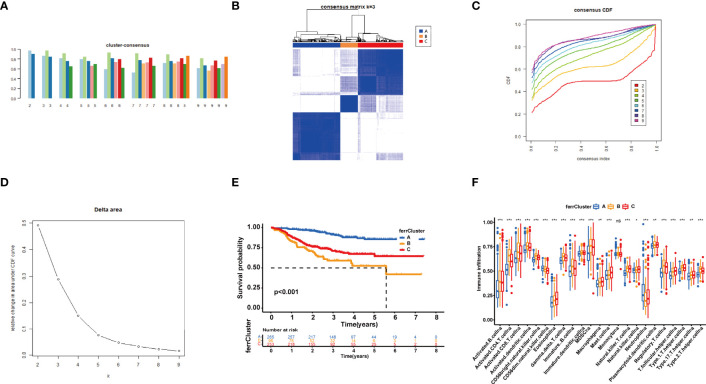
Consensus clustering of 18 ferroptosis-related genes (FRGs) identified three clusters of patients. **(A)** The tracking plot for k = 2 to k = 9. **(B)** The heatmap for K = 3. **(C)** Consensus clustering cumulative distribution function (CDF) with k = 2 to k = 9. **(D)** Relative change in area under CDF curve for k = 2–9. **(E)** Kaplan–Meier (K-M) curve of the survival difference among clusters 1–3. **(F)** Single-sample gene set enrichment analysis of immune status among three ferrClusters. ns, P>0.05. *, P < 0.05; **, P < 0.01; ***, P < 0.001.

On the basis of the expression of 18 FRGs in the signature, we employed the “Partition Around Medoids” algorithm, along with Pearson’s distance to estimate similarity among patients to identify three clusters. We noticed that K = 3 seemed to be an optimal selection by clustering variable (k) increasing from 2 to 9, in which the intergroup correlations were the lowest and the intragroup correlations were the greatest (**Figure 6B**), indicating the optimal clustering stability of the three molecular phenotypes. The consensus cumulative distribution function (CDF) diagram showed that when k = 3, distribution reached an approximate maximum (**Figure 6C**), implying robust clustering for all samples (**Figure 6A**). The delta area plot depicts the relative change compared to k − 1 showing that the delta area was optimum when k = 3 (**Figure 6D**). Prognostic analysis of the three clusters revealed that patients in ferrCluster A were the least likely to relapse, while in ferrCluster B, they were the most likely to relapse (**Figure 6E**).

We then performed an ssGSEA to quantify the scores of various immune cell subpopulations to further compare the differences in the number of immune cells among the three types of ferrClusters. The results indicated that the contents of monocyte cells were not significantly different. The proportion of immune cells was significantly different among the three clusters. Contents of nearly all types of immune cells in ferrCluster A seemed to be the poorest. The levels of activated CD4+ T cells, CD8+ T cells, dendritic cells, CD56 bright and dim NK cells, γδ-T cells, Tregs, and T helper cells were relatively the highest in ferrCluster B. Hence, we could draw the conclusion that ferrCluster A was a type of immune failure, ferrCluster B was a type of immune-activated characterized by T-cell subset enrichment, and ferrCluster C was a type of immune-activated characterized by B-cell subset enrichment (**Figure 6F**). These results indicated that the FRGs play key roles in immune cell infiltration and characteristic tumor immune microenvironment (TME) formation and affect the prognosis of BRCA patients.

### Development of ferrScore to Quantify Individual Ferroptosis Pattern

**Figure 7:**
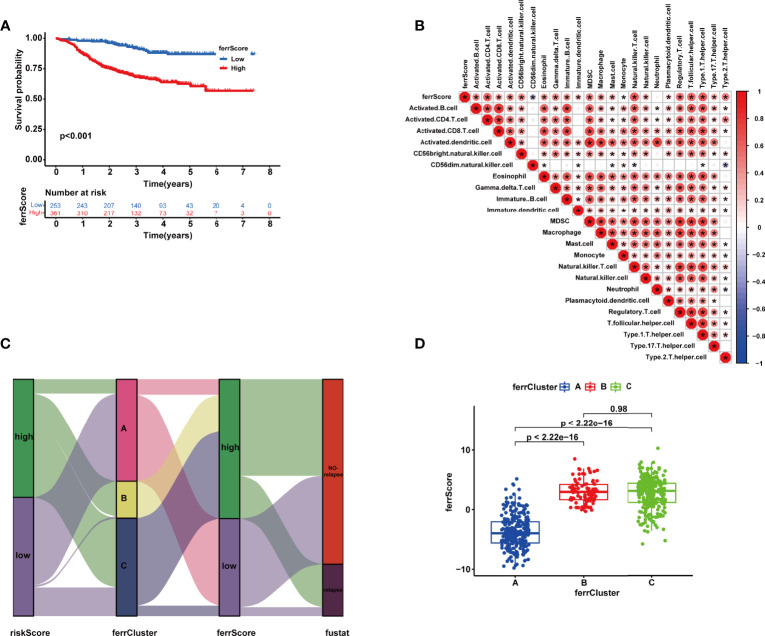
**(A)** Kaplan–Meier (K-M) curve of the survival difference between high and low ferrScore groups. **(B)** Correlation between immune cells and ferrScore. **(C)** Alluvial diagram of riskScore group, ferrCluster group, ferrScore group, and relapse-free status. **(D)** Correlation between ferrCluster and ferrScore *, P < 0.05.

With a view to the individual heterogeneity and complexity of BRCA patients, we calculated ferrScore to assess the ferroptosis pattern of each patient based on the PCA on the 18 FRGs in the model. The scoring framework was defined as ferrScore = PC1 + PC2 to quantify individual ferroptosis patterns of BRCA patients (36), further facilitating precise treatment. As indicated from the K-M curve, patients with lower ferrScore had a lower probability of relapse (**Figure 7A**). The ferrScore was closely related to immune cells (**Figure 7B**). We also observed that ferrScores of patients in ferrCluster A were significantly lower than those in ferrCluster B and C, while there was no significant difference between ferrClusters B and C (**Figure 7D**). The Sankey diagram shows the attribute changes in riskScore, ferrCluster, ferrScore, and recurrence status, indicating that the higher the riskScore and ferrScore, the higher the risk of relapse after receiving NAC (**Figure 7C**). The above results enriched treatment strategies for BRCA patients not only in targeted therapy and chemotherapy but also in immunotherapy. At last, the expression of CTLA4 was examined to elucidate a potential response to immunotherapy, and the high ferrScore group showed relatively high levels of expression (p = 8.6e−11, **Figure 7B**).

## Discussion

Ferroptosis is a newfound programmed cell death pattern distinguished from traditional cell death such as apoptosis, necrosis, and autophagy (37). Accumulating evidence demonstrated that dysregulated expression and genetic variations of FRGs were closely related to cell death, tumor carcinogenesis, and progression (22, 38).

TME is a cradle for tumorigenesis and cancer progression, in which immune infiltrating cells affect therapeutic outcomes (39). The relationships between TME infiltration immune cells and ferroptosis modifications have become a hotspot in the mechanism of tumorigenesis and development (40, 41). MIF secreted by nasopharyngeal carcinoma could suppress ferroptosis of macrophages and then increase the rate of metastasis (42). BEBT-908 induces immunogenic ferroptosis to potentiate cancer immune checkpoint therapy (43). SCD1 and FABP4 could drive ferroptosis, thereby leading to tumor resistance (44). Ferroptotic cells could also release chemotaxis to interact with immune cells, such as CD + T cells, and then modulate the anticancer immunity (45).

High-throughput genomic studies provided cutting-edge sights into the molecular mechanisms and identified new potential targets of breast cancer. Our research developed and verified a stepwise multivariate Cox regression model including 18 FRGs using LASSO and multivariate Cox regression for removing redundant factors to forecast the RFS of individual patients in GSE25055. The expression of FRGs in the signature was higher in BRCA tissues than in adjacent normal tissues, which was verified in several breast cancer cell lines using real-time PCR. Meanwhile, CNVs and mutation frequencies of FRGs were prevalent. Internal and external validations exhibited an excellent ability to predict the prognosis of BRCA patients. Specifically, a higher riskScore indicated a higher rate of recurrence. Moreover, riskScore was associated closely with clinicopathological features.

With a view to the significance of the immune system in antiviral and antitumor responses, we calculated the proportion of different types of tumor-infiltrating immune cells in BRCA using CIBERSORT and used ESTIMATE to explore IS, SS, and tumor purity. Higher SSs and ISs were observed in high-risk patients, leading to an unfavorable prognosis, which was consistent with a line of evidence from previous research (46, 47).

Extensive interest in cancer immunotherapy is reported according to the clinical importance of CTLA-4 and PD-1/PD-L1 in immune checkpoint therapies (48). The main immune checkpoints for breast cancer include CTLA-4, PD-1/PD-L1, lymphocyte activation gene 3 (LAG-3), T-cell immunoglobulin domain and mucin 3 (TIM-3), and other molecules (49). Clinical trials like SOLTI-1503 PROMETEO TRIAL (50), KEYNOTE-086 (51), NIMBUS (52), KEYNOTE-173 (53), and KEYNOTE-522 (54) showed that immunological checkpoint inhibitors have made significant progress in breast cancer immunotherapy, which is expected to become a new treatment for breast cancer.

Furthermore, for the purpose of exploring the response to chemotherapy sensitivity of patients, we calculated the IC50 value. The sensitivities of chemotherapeutic drugs widely used in BRCA showed a significant difference between the two groups.

In accordance with the expression matrix of the 18 FRGs in the signature, we identified three ferroptosis-related molecular clusters *via* consensus clustering analysis. The rate of relapse was significantly different among the three clusters. ssGSEA identified that the three ferrClusters as three immune types of immune failure, immune-activated characterized by T-cell subset enrichment, and immune-activated characterized by B-cell subset enrichment.

Inevitably, numerous limitations of our study should be included in the consideration. First, although our conclusion came through internal and external validation in TCGA, GSE25055, GSE25065, and GSE16446 cohorts, when it comes to its clinical application, caution is advised. Multicenter large-scale prospective clinical studies were needed rather than only retrospective data from public open databases to verify the signature. Second, the expression matrix of patients in GSE25055 and GSE25065 was extracted *via* platform GPL96 [HG-U133A] Affymetrix Human Genome U133A Array in 2010, which only included 12,549 genes, while GPL570 [HG-U133_Plus_2] Affymetrix Human Genome U133 Plus 2.0 Array for GSE16446 contained 21,655 genes. Due to the relatively small number of detectable genes, bias may be amplified. Finally, detailed molecular mechanisms in the BRCA of the FRGs in the signature had not been fully revealed. Further in-depth studies were required to confirm relationships between FRGs and tumor microenvironment, and between ferroptosis and chemoresistance.

## Conclusion

In brief, we constructed a novel FRG signature and identified three molecular subtypes for predicting the RFS of BRCA patients, which could predict the immune status of the tumor microenvironment and RFS of patients. It is worth noting that our conclusions provided more clues for the rational choices of chemotherapeutic drugs for patients with BRCA, provided a new immunological perspective and a new basis for immunotherapy of BRCA in the clinic, and had the potential possibility to coach and guide individualized healthcare decisions.

## Data Availability Statement

The expression and clinical datasets presented in this study are available in databases of TCGA and Gene Expression Omnibus (GSE25055, GSE25065 and GSE16446), and the copy number variation data are presented in the University of California, Santa Cruz (UCSC) website https://xenabrowser.net/datapages/?dataset=TCGA-BRCA.gistic.tsv&host=https%3A%2F%2Fgdc.xenahubs.net&removeHub=https%3A%2F%2Fxena.treehouse.gi.ucsc.edu%3A443.

## Author Contributions

XM and QX substantially contributed to the conception of the work. YX, QZ, and TZ contributed to the data collection. YX, YD, and QX wrote the manuscript. QZ and YD helped to perform the enrichment and network analysis. YX, YD, and XM drafted and revised the manuscript. All authors contributed to the article and approved the submitted version.

## Funding

This research was supported by the Zhejiang Provincial Natural Science Foundation of China (Grant No. LQ21H200007), Zhejiang Provincial Ministry Medical and Health Co-construction Major Project (Grant No. 20214355173), and National Natural Science Foundation of China (Grant No. 81973861).

## Conflict of Interest

The authors declare that the research was conducted in the absence of any commercial or financial relationships that could be construed as a potential conflict of interest.

## Publisher’s Note

All claims expressed in this article are solely those of the authors and do not necessarily represent those of their affiliated organizations, or those of the publisher, the editors and the reviewers. Any product that may be evaluated in this article, or claim that may be made by its manufacturer, is not guaranteed or endorsed by the publisher.

## Supplementary Material

Supplementary Table 1 Ferroptosis suppressor, driver and marker genes from FerrDB database.

## Abbreviations

GEO, Gene Expression Omnibus; TCGA, The Cancer Genome Atlas; NAC, neoadjuvant chemotherapy; CNV, copy number variation; UCSC, University of California, Santa Cruz; ROC, receiver operating characteristic; GDSC, Genomics of Drug Sensitivity in Cancer; IC50, half-maximal inhibitory concentration; AUC, area under the curve; RFS, recurrence-free survival; FRG, ferroptosis-related gene; IS, immune score; SS, stromal score; ES, estimate score; ssGSEA, single-sample gene set enrichment analysis; TIME, tumor immune microenvironment; PCA, principal component analysis; ICI, immune checkpoint inhibitor.
